# Influence of Ultrafine 2CaO·SiO_2_ Powder on Hydration Properties of Reactive Powder Concrete

**DOI:** 10.3390/ma8095300

**Published:** 2015-09-17

**Authors:** Hongfang Sun, Zishanshan Li, Shazim Ali Memon, Qiwu Zhang, Yaocheng Wang, Bing Liu, Weiting Xu, Feng Xing

**Affiliations:** 1 Guangdong Province Key Laboratory of Durability for Marine Civil Engineering, School of Civil Engineering, Shenzhen University, Shenzhen 518060, Guangdong, China; E-Mails: sunhf03@szu.edu.cn (H.S.); lzssaus@gmail.com (Z.L.); wangycszu@gmail.com (Y.W.); liubing0708@szu.edu.cn (B.L.); 2 Department of Civil Engineering, COMSATS Institute of Information Technology, Abbottabad 22010, Pakistan; E-Mail: shazimalimemon@gmail.com; 3 Department of Civil Engineering, Chongqing University, Chongqing 400044, China; E-Mail: 20125595@cqu.edu.cn

**Keywords:** ultrafine, hydration, reactive powder concrete, microstructure, compressive strength

## Abstract

In this research, we assessed the influence of an ultrafine 2CaO·SiO_2_ powder on the hydration properties of a reactive powder concrete system. The ultrafine powder was manufactured through chemical combustion method. The morphology of ultrafine powder and the development of hydration products in the cement paste prepared with ultrafine powder were investigated by scanning electron microscopy (SEM), mineralogical composition were determined by X-ray diffraction, while the heat release characteristics up to the age of 3 days were investigated by calorimetry. Moreover, the properties of cementitious system in fresh and hardened state (setting time, drying shrinkage, and compressive strength) with 5% ordinary Portland cement replaced by ultrafine powder were evaluated. From SEM micrographs, the particle size of ultrafine powder was found to be up to several hundred nanometers. The hydration product started formulating at the age of 3 days due to slow reacting nature of belitic 2CaO·SiO_2_. The initial and final setting times were prolonged and no significant difference in drying shrinkage was observed when 5% ordinary Portland cement was replaced by ultrafine powder. Moreover, in comparison to control reactive powder concrete, the reactive powder concrete containing ultrafine powder showed improvement in compressive strength at and above 7 days of testing. Based on above, it can be concluded that the manufactured ultrafine 2CaO·SiO_2_ powder has the potential to improve the performance of a reactive powder cementitious system.

## 1. Introduction

Reactive powder concrete (RPC) is a cement-based composite known for its superior mechanical and durability performance over conventional concrete [1,2]. The superior performance of RPC is achieved by using micro-structural engineering approach, such as elimination of coarse aggregate, using low water to cementitious material ratio, lowering the CaO to SiO_2_ ratio, and incorporating steel micro-fibers [3,4]. The optimization of the gradation and arrangement of the inert particles as well as the design of the hydraulic reactive components are the key features of RPC for properties enhancement. The ultrafine reactive powders are incorporated as partial cement replacement to increase packing (either by filler effect or chemical reaction such as pozzolanic reaction and conventional hydration), which consequently improves the overall performance of RPC. The ultrafine reactive powders reported in literature [3,5,6,7,8] usually include silica fume, slag, and fly ash, *etc.* However, researchers are still seeking alternative ultrafine reactive powder for improving the performance of RPC [9,10,11,12,13].

As an alternative [14], a combustion approach was proposed to synthesize ultrafine construction materials. This approach has been widely used in producing advanced ceramic materials [15,16,17,18]. In this approach, raw materials (such as limestone, clay, and aluminum nitrate, *etc.*) were mixed with stoichiometric amounts of fuel (carbohydrazide, citric acid, and glycine nitrate, *etc.*) and heated to a certain temperature (usually several hundred degrees Celsius) to initiate combustion. After that, the produced clinker with slight grinding is collected for further analysis. The ultrafine material manufactured by this method has been reported to react quickly [14,19]. However, as per the author’s knowledge, the literature about the synthesis process, the microstructure of the resulting material and how these, in turn, influence the performance of RPC, is scarce. Similar combustion method was utilized by Halim *et al.* [20] to produce calcium silicate-based nano-particles with organic compounds. However, the authors only characterized the produced nano-particles and did not check its influence on RPC.

Thus, in this research, we manufactured an ultrafine 2CaO·SiO_2_ powder (UFP) through chemical combustion process and studied its influence on RPC. The morphology and heat release characteristics of UFP were compared with that of the conventional cement. The development of hydration products in cement paste containing UFP was assessed. Moreover, the influence on properties (setting time, shrinkage, and compressive strength) of RPC with 5% cement replaced by UFP was evaluated.

## 2. Experimental Details

### 2.1. Materials

For the synthesis of the UFP material, limestone and silica fume (SF) were used as raw materials while urea and nitric acid were used as fuel source. The limestone (99% purity CaCO_3_), urea (99% purity CO(NH_2_)_2_), and concentrated nitric acid (component content: 65%–68%) were purchased from Xilong Chemical Co., Ltd. (Guangzhou, China) while SF was obtained from BENNO Industrial Co. Ltd. (Shenzhen, China).

In order to examine the influence of UFP on RPC, ordinary Portland cement (OPC), silica fume (SF), superplastisizer (SP) and fine aggregates were used. OPC with strength grade of 42.5 was from Hailuo Industry Co. Ltd. (Anhui, China), while silica fume was obtained from BENNO Industrial Co. Ltd. (Shenzhen, China). The physical and chemical properties of OPC and SF are enlisted in Table 1. Furthermore, standard quartz sand (China ISO Standard Sand Co., Ltd., Xiamen, China) having fineness modulus of 3.02 and specific gravity of 2.61 was used as fine aggregate while Sika ViscoCrete produced by Sika corporation (Guangzhou, China) was used as superplasticizer.

**Table 1:** Chemical composition and physical properties of ordinary Portland cement (OPC) and silica fume (SF).

Chemical Composition	OPC (%)	SF (%)
SiO_2_	21.35	95.55
Al_2_O_3_	5.51	0.32
Fe_2_O_3_	4.56	0.41
SO_3_	1.85	-
CaO	64.38	0.19
MgO	0.78	0.30
Na_2_O	0.07	0.21
K_2_O	0.65	0.50
Loss on ignition (LOI)	0.85	2.68
Physical properties	-	-
BET surface area (m^2^/g)	2.85	18–28
Mean particle size (μm)	15.50	0.1–0.3

### 2.2. Synthesis Procedure

**Figure 1:**
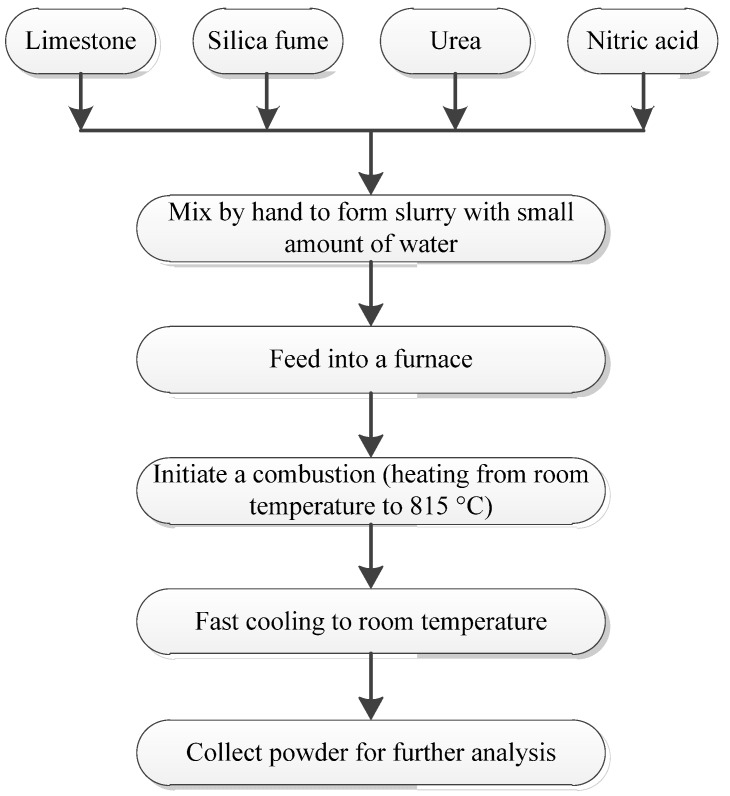
Flow chart—Synthesis of ultrafine 2CaO·SiO_2_ powder (UFP).

The flow chart for synthesizing of UFP is shown in Figure 1. Limestone and SF powders were mixed with the urea, nitric acid, and a certain amount of water according to the mix proportion given in Table 2. In order to form slurry, the mixture was stirred by hand with a stirrer for 1 min to form slurry. Thereafter, the slurry was heated at a heating rate of 20 °C/min in a furnace to reach 815 °C. The combustion lasted for approximately 1 min. Finally, the sample was fast cooled outside the furnace to room temperature and the collected sample was characterized.

**Table 2:** Mix proportion for synthesizing ultrafine 2CaO·SiO_2_ powder (UFP).

Ingredients	Limestone	SF	Urea	Nitric Acid	Water
Fraction (%)	10.92	4.16	47.21	23.33	14.38

### 2.3. Characterization Techniques

#### 2.3.1. Scanning Electron Microscopy (SEM)

The SEM analysis was performed on FEI Quanta 250 microscope (FEI Inc., Hillsboro, OR, USA) with a field emission gun working at 15 kV. The chamber pressure for secondary electron (SE) observation was 300 Pa (environmental scanning electron microscopy (ESEM) mode) and it was 3 × 10^−4^ Pa (high vacuum mode) for backscattering electron (BSE) observation. The surface morphology of unhydrated UFP and OPC was observed directly with the SE while the cross-section of UFP and OPC powder was observed using BSE detector. For sample preparation for BSE observation, approximately 4 g of the unhydrated powder was mixed with 3 g of epoxy resin so as to form a viscous paste. The mixture was then placed in a plastic mold (10 mm diameter and 55 mm height) and cured at room temperature for 24 h. Thereafter, the cured specimens were cut by a low-speed diamond saw and polished using the procedure mentioned in [21]. Finally, before loading the specimen into the SEM chamber, the specimens were gold coated so as to form a conductive surface.

In order to study the hydration of UFP, the powder was mixed by hand (approximately 90 s) with deionized water. The water to cement ratio was kept at 1.2. At the desired age of testing, the microstructure of paste was observed with ESEM so as to avoid the artifact caused by drying or coating under high vacuum mode.

#### 2.3.2. X-ray Diffraction (XRD)

The mineralogical analysis of UFP was carried out by XRD on Bruker D8 instrument (Karlsruhe, Germany) with a CuKα source at 40 kV and 200 mA while the semi-quantitative analysis of different phases present in UFP was performed using a Jade program.

#### 2.3.3. Calorimetry

The isothermal calorimetry was performed up to approximately 3 days using a ToniCal Trio 7338 instrument (Toni Technik, Zwick/Roell Group, Berlin, Germany). The step by step procedure adopted for testing is as follows; (a) Weigh 3 g of UFP and put into a test tube; (b) Draw 3.6 g water into a syringe which is coupled with the test tube; (c) Put the system into the chamber; (d) When the temperature of the chamber reaches equilibrium (*i.e*., the baseline is flat), empty the syringe and allow water to make contact with UFP; (e) Collect calorimetry data until the desired age of testing.

#### 2.3.4. Initial and Final Setting Time

In order to determine the influence of UFP on the setting rate of RPC, the initial and final setting time of RPC control and blended RPC-UFP (95% OPC + 5% UFP) pastes (Table 3) were monitored by following the procedure mentioned in ASTM C403 [22].

**Table 3:** Mixture proportion for setting time test. SP: superplastisizer.

Specimens	OPC (g)	UFP (g)	SF (g)	Water (g)	SP (g)
RPC control paste	100	0	10	1.6	21
RPC-UFP paste	95	5	10	1.6	21

#### 2.3.5. Drying Shrinkage and Compressive Strength

The details of mixture proportion for drying shrinkage and compressive strength tests are shown in Table 4. For all the specimens, the water to binder (OPC + UFP) ratio was maintained at 0.21 while the sand to binder (OPC + UFP) ratio was 1:1 by mass.

**Table 4:** Mixture proportion for drying shrinkage and compressive strength tests.

Specimens	OPC (g)	UFP (g)	SF (g)	Sand (g)	Water (g)	SP (g)
RPC control	1000	0	100	1000	210	16
RPC-UFP	900	100	100	1000	210	16

The mixing of samples was carried out in Hobart mixer. At first, the materials except sand were mixed at slow speed for 30 s followed by mixing at fast speed for approximately 4 min. Thereafter, the sand was added and the mixing was continued at fast speed for another 5.5 min.

After mixing, the mixture was placed into 40 × 40 × 80 mm^3^ mold and cured for 3 days. After that, the specimen were demolded and placed into different curing rooms (20 ± 2 °C and 60% relative humidity (RH) for drying shrinkage test and 20 ± 2 °C and 90% RH for compressive strength test) until the day of testing.

To assess the rate of deformation, the drying shrinkage was measured at the age of 3, 7, 14, 28, and 45 days according to ASTM C596 [23]. Three samples were tested for obtaining the average value of the drying shrinkage by using Δ*L*/*L* × 100%, where *L* is the original length of sample while Δ*L* is the dimensional variations in length due to shrinkage at the desired ages.

The compressive strength of samples was determined at the age of 3, 7, 14, 28, and 45 days using a YAW-300B compression machine (Jinan Shidai Shijin Testing Machine Group Co., Ltd., Jinan, China). Three samples (40 × 40 × 160 mm^3^) were tested at each age and the average value was reported.

## 3. Results and Discussion

### 3.1. Morphology of Unhydrated UFP

**Figure 2:**
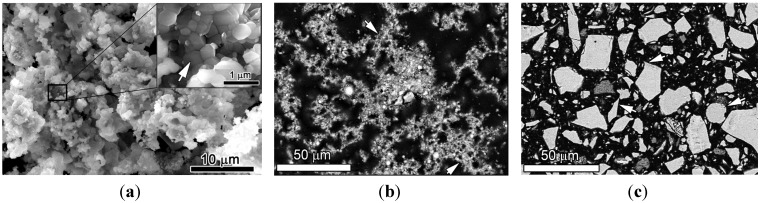
The morphology of UFP and ordinary Portland cement (OPC). (**a**) Secondary electron (SE) image of UFP; (**b**) Backscattering electron (BSE) image of UFP; (**c**) BSE image of OPC.

The SEM images of as-prepared UFP as well as OPC powders are shown in Figure 2. It can be seen that UFP consists of agglomerated round fine particles with porous structure (arrows in Figure 2a,b). The particle size is up to several hundred nanometers as seen in the enlarged inset of Figure 2a. In comparison to UFP, the shapes of commercial OPC particles appear to be random, *i.e*., round, elongated, and angular (arrows in Figure 2c). The difference in shape of OPC particles arises from grinding of clinker nodules to cement powder, the clinker texture and phase composition, and different grindability of the constituent phases [24,25]. The size of UFP is in hundreds of nanometers while the size of the OPC particle is up to tens of micrometers. The difference in size between UFP and OPC may be related to the combustion method used in this study. Furthermore, the UFP material is finer than the raw materials used to produce the UFP due to the gas escaping as reported in literature [16].

### 3.2. Mineralogical Analysis of UFP

**Figure 3:**
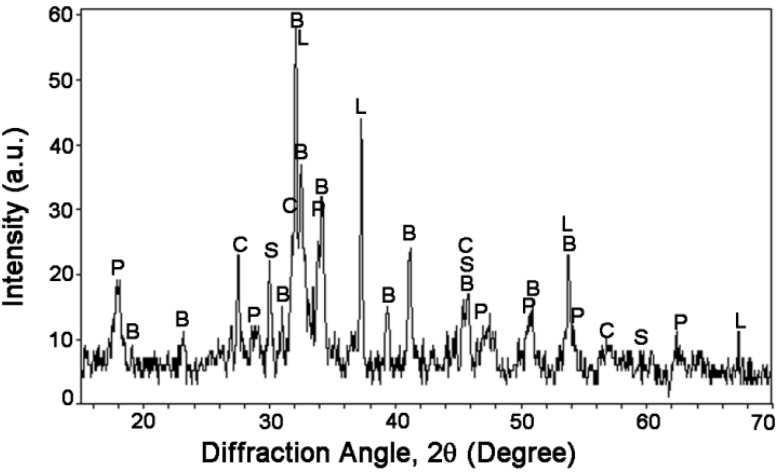
X-ray diffraction (XRD) diffractogram of ultrafine 2CaO·SiO_2_ powder (UFP). B-2CaO·SiO_2_ (larnite), P-Ca(OH)_2_, L-CaO, S-SiO_2_, C-CaO·SiO_2_.

The mineralogical analysis of UFP was conducted with XRD technique while the semi-quantitative analysis (Rietveld fitting) was performed on diffractogram (Figure 3). The results of semi-quantitative analysis are presented in Table 5. 2CaO·SiO_2_ (larnite, β-C_2_S) was found to be the dominant crystalline phase with a mass fraction of 58.3%. The presence of Ca(OH)_2_ (portlandite, CH) with a mass fraction of 23.7% may have resulted from the hydration of 2CaO·SiO_2_ with the water residue present in the raw materials. Moreover, the presence of CaO (lime, mass fraction 8.7%) from the decomposition of limestone may affect the hydration of cement paste at the very early age. Besides, UFP also contained crystalline phases (SiO_2_ (quartz, mass fraction 9.1%) and CaO·SiO_2_ (wollastonite, mass fraction 0.2%)), which are hydraulically in-active and may have less influence on hydration of cement paste. For comparison purpose, the mineralogical composition of OPC was also estimated from Table 1 through Bogue equations [26]. The OPC mainly consisted of 51.1% 3CaO·SiO_2_ (alite, C_3_S), 22.7% 2CaO·SiO_2_ (larnite, β-C_2_S), 6.9% 3CaO·Al_2_O_3_ (aluminates, C_3_A) and 13.7% 4CaO Al_2_O_3_·Fe_2_O_3_ (ferrite, C_4_AF). In comparison to OPC, the UFP is hydraulically less reactive since both 3CaO·SiO_2_ and 3CaO·Al_2_O_3_ in OPC hydrates much faster than 2CaO·SiO_2_ in UFP. However, UFP has finer particles than OPC, which may accelerate the hydration of UFP.

**Table 5:** Composition of UFP from X-ray diffraction (XRD).

Composition	2CaO·SiO_2_	Ca(OH)_2_	CaO	SiO_2_	CaO·SiO_2_
Fraction (%)	58.3	23.7	8.7	9.1	0.2

### 3.3. Hydration of UFP

**Figure 4:**
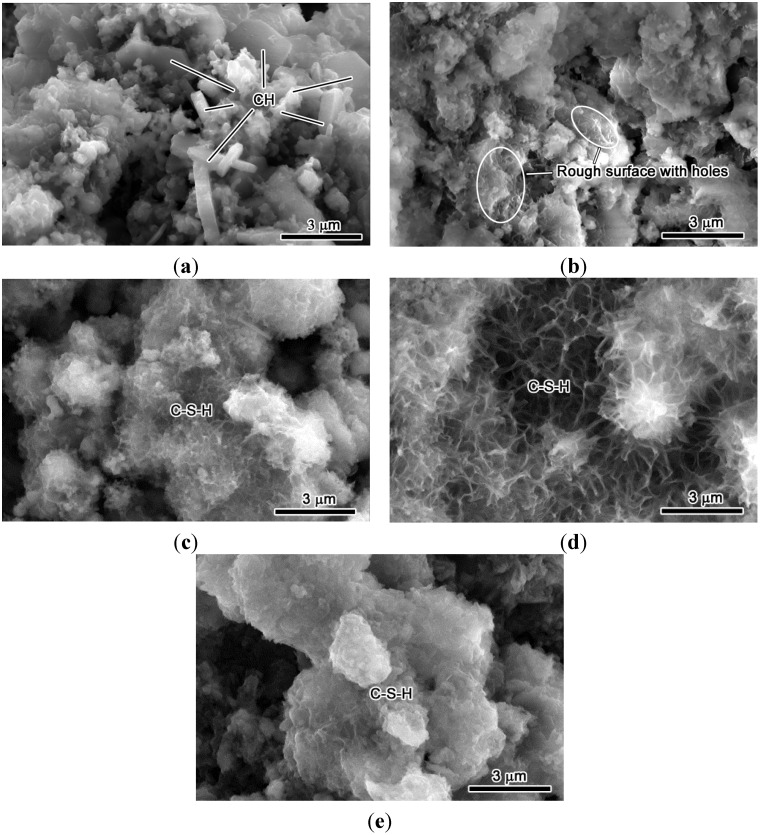
Environmental scanning electron microscopy (ESEM) observation of hydration process of UFP paste. The 1 (**a**), 3 (**b**), 7 (**c**), 14 (**d**) and 28-day (**e**) results of UFP paste are shown, respectively.

The microstructure of UFP paste up to the age of 28 days was observed through ESEM so as see the changes in the hydration products. The micrographs of hydrated UFP paste at various ages are shown in Figure 4. At the age of 1 day, no significant hydration was observed due to the slow-reacting nature of belite minerals of 2CaO·SiO_2_ (Figure 4a) except the appearance of CH crystals, which is supposed to be from the hydration of CaO in UFP (Table 5). At the age of 3 days, the hydration of 2CaO·SiO_2_ has initiated with a sign of rough surface consisting of series of holes (Figure 4b). The micrograph at the age of 7 days showed foil-like features, which is supposed to be the hydration product of 2CaO·SiO_2_ ((CaO)*_x_*-SiO_2_-(H_2_O)*_y_*, abbreviated as C-S-H) (Figure 4c). At the age of 14 days, the C-S-H foils grew and covered the entire surface of UFP cement paste (Figure 4d). This foil-like morphology indicates the presence of low Ca/Si (<1.5) in C-S-H [27]. Finally, the feature of C-S-H at the age of 28 days transformed from loose (at 14 days) to dense (Figure 4e), indicating the formation of a compact structure due to the hydration of UFP and therefore contributing to the development of strength.

### 3.4. Calorimetry

**Figure 5:**
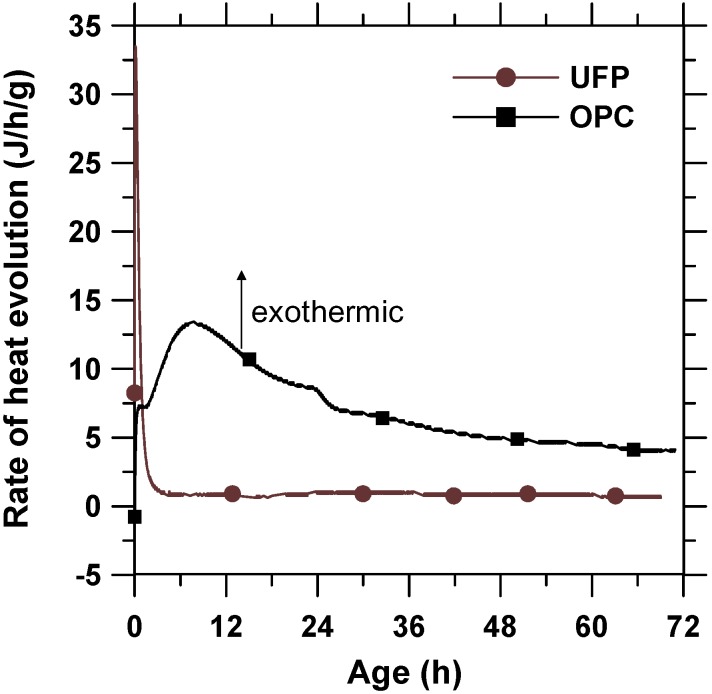
Heat release profile of UFP and OPC pastes during the first 3 days.

The hydration of cementitious materials is usually accompanied by an exothermic process. Therefore, the heat release behavior of UFP and OPC up to the age of 3 days was studied through calorimetry so as to compare the hydration rate of UFP with OPC. The heat release profile of UFP and OPC pastes are shown in Figure 5. The initial peak of UFP is much higher than that of OPC due to much higher content of CaO in UFP than in OPC. Moreover, it is known that the hydration of CaO is a fast exothermic reaction. Thereafter, the heat release of UFP remained low indicating slow hydration during the first 3 days. The results of calorimetry were consistent with the observations made in Section 3.3, where minor sign of hydration of 2CaO·SiO_2_ was observed at the age of 3 days and had not contributed much to the heat release yet.

### 3.5. Initial and Final Setting Time

The initial and final setting time of RPC control and RPC-UFP paste was tested to assess the influence of UFP on setting rate of RPC. The results are listed in Table 6. It can be seen that the RPC control paste has an initial setting time of 4.20 h and final setting time of 5.28 h. The addition of UFP (5% replacement of OPC) delayed the initial setting time by 0.30 h and the final setting time by 0.97 h due to the existence of slow reacting nature of 2CaO·SiO_2_ in UFP.

**Table 6:** Initial and final setting time of reactive powder concrete (RPC) control and RPC-UFP pastes.

Specimens	Initial Setting Time (h)	Final Setting Time (h)
RPC control paste	4.20	5.28
RPC-UFP paste	4.50	6.25

### 3.6. Drying Shrinkage

**Figure 6:**
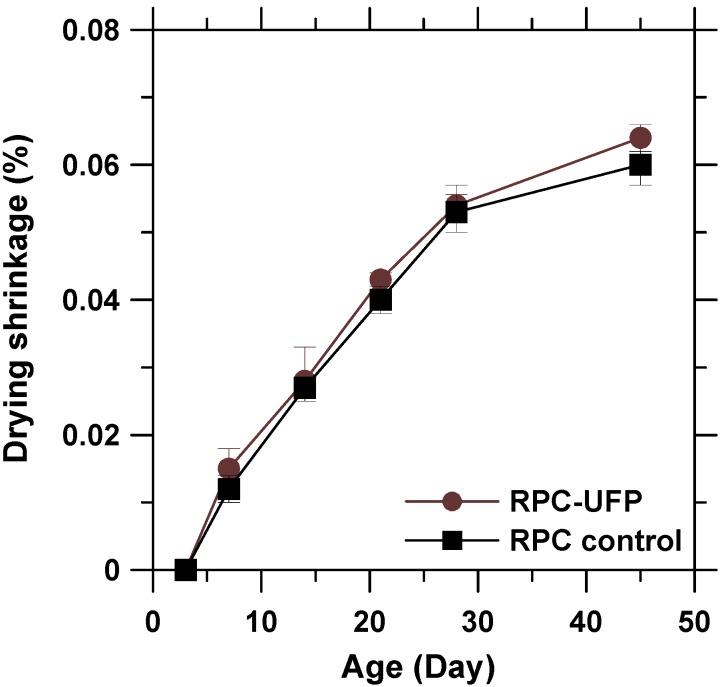
Drying shrinkage of reactive powder concrete (RPC) control and RPC-UFP.

The drying shrinkage of RPC-UFP was tested to reflect the influence of UFP on volume stability. The results are shown in Figure 6. In comparison to RPC control, the RPC-UFP showed almost equal or little bit higher shrinkage values at all the ages of testing. The maximum difference in drying shrinkage between RPC control and RPC-UFP was obtained at the age of 45 days and was found to be less than 8%. Therefore, it can be deduced that the addition of UFP has no significant influence on the drying shrinkage of the resulting cementitious system. It is worth mentioning here that the drying shrinkage is mainly related to evaporation of adsorbed water in large pores and the water in small capillary pores in hydrated cement paste [28]. The result indicated that the addition of UFP with a small amount of 5% had insignificant influence on the pore structure in RPC.

### 3.7. Compressive Strength

**Figure 7:**
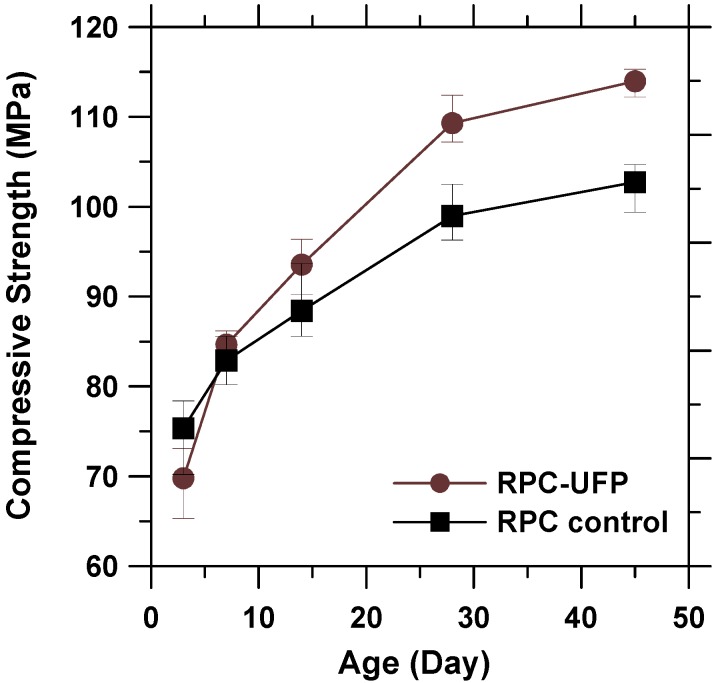
Compressive strength of RPC control and RPC-UFP.

The compressive strength results of RPC control and RPC-UFP are shown in Figure 7. It can be seen that the RPC-UFP mix showed lower compressive strength at the age of 3 days. As mentioned earlier, this may be due to the slow reacting nature of 2CaO·SiO_2_. However, the RPC-UFP mix showed higher values of compressive strength at and above 7 days of testing. At the age of 45 days, the RPC-UFP mix showed compressive strength value of 114 MPa, which is 11% higher than RPC control mix. The reason behind the increase in compressive strength of RPC-UFP mix may be due to the filler effect of the ultrafine UFP material and the pozzolanic reaction between silica fume and CH in UFP.

## 4. Conclusions

In this research, an ultrafine 2CaO·SiO_2_ powder was manufactured using the chemical combustion method and its potential application in RPC was investigated. The main results are as follows:

1. From ESEM, the 2CaO·SiO_2_ powder was found to be round in shape, porous in nature, and its size was up to hundred nanometers. XRD data showed that 2CaO·SiO_2_ (larnite) with mass fraction of approximately 60% was found to be the dominant composition of UFP.
2. From ESEM micrographs, it was found that the hydration of UFP paste started at the age of 3 days and the microstructure transformed into dense structure of C-S-H at the age of 28 days. The rate of hydration was also verified by calorimetry, which showed that the hydration of UFP at the early age is much slower than that of OPC due to the slow reaction nature of 2CaO·SiO_2_.
3. In comparison to RPC control paste, the initial and final setting time of RPC-UFP paste delayed by 0.3 and 0.97 h due to the presence of 2CaO·SiO_2_ in UFP. No significant difference in drying shrinkage values was observed in between RPC control and RPC-UFP mix. In comparison to RPC control, the RPC-UFP mix showed improvement in compressive strength at and above 7 days of testing.

Based on the above, it can be concluded that the manufactured UFP can be used to produce a reactive powder cementitious system. Moreover, for future work, it is recommended to optimize the replacement level of OPC by UFP.
